# Clinical trajectory of intraductal papillary mucinous neoplasms progressing to pancreatic carcinomas during long-term surveillance: a prospective series of 100 carcinoma cases

**DOI:** 10.1007/s00535-023-02028-0

**Published:** 2023-07-29

**Authors:** Hiroki Oyama, Tsuyoshi Hamada, Yousuke Nakai, Mariko Tanaka, Go Endo, Ryunosuke Hakuta, Kota Ishida, Kazunaga Ishigaki, Sachiko Kanai, Kohei Kurihara, Tomotaka Saito, Tatsuya Sato, Tatsunori Suzuki, Yukari Suzuki, Shinya Takaoka, Shuichi Tange, Yurie Tokito, Naminatsu Takahara, Tetsuo Ushiku, Mitsuhiro Fujishiro

**Affiliations:** 1 Department of Gastroenterology, Graduate School of Medicine, The University of Tokyo, Tokyo, Japan; 2 Department of Hepato-Biliary-Pancreatic Medicine, The Cancer Institute Hospital of Japanese Foundation for Cancer Research, Tokyo, Japan; 3 Department of Endoscopy and Endoscopic Surgery, The University of Tokyo Hospital, 7-3-1 Hongo, Bunkyo City, Tokyo, 113-8655 Japan; 4 Department of Pathology, Graduate School of Medicine, The University of Tokyo, Tokyo, Japan

**Keywords:** Carcinogenesis, Cohort studies, Pancreatic cyst, Pancreatic neoplasms, Risk factors

## Abstract

**Background:**

Trajectories of serological and morphological signatures have not been documented in pancreatic carcinogenesis related to intraductal papillary mucinous neoplasms (IPMNs).

**Methods:**

Using a prospective cohort of 3437 IPMN patients, we identified 100 IPMN patients who developed pancreatic carcinomas during long-term surveillance. We examined serial changes of blood markers (carbohydrate antigen 19-9 [CA19-9], hemoglobin A1c [HbA1c], and pancreatic enzymes) and morphological features (worrisome features and high-risk stigmata) during the prediagnostic period of pancreatic carcinomas, overall and by carcinoma types (IPMN-derived vs. concomitant pancreatic carcinomas).

**Results:**

CA19-9 elevation was observed in 39 patients and was associated with a metastatic stage. Compared to IPMN-derived carcinomas, concomitant carcinomas were more likely to represent CA19-9 elevation (60% vs. 30%, respectively; *P* = 0.005). HbA1c levels elevated only in 3 patients. Pancreatic enzyme elevation was observed in 18 patients with no differences in frequencies between the carcinoma types. All patients with elevated levels of blood markers had positive findings on cross-sectional imaging. High-risk stigmata or worrisome features were observed in all patients but one with concomitant carcinoma. The most common types of worrisome features were the main pancreatic duct dilatation and CA19-9 elevation in IPMN-derived and concomitant carcinomas, respectively. Compared to IPMN-derived carcinomas, concomitant carcinomas were less likely to harbor high-risk stigmata (16% vs. 86%, respectively; *P* < 0.001).

**Conclusions:**

The usefulness of currently available blood biomarkers was limited in early detection of pancreatic carcinomas related to IPMNs. Morphological alterations were well correlated with long-term risk of IPMN-derived carcinomas, but not with that of concomitant carcinomas.

**Supplementary Information:**

The online version contains supplementary material available at 10.1007/s00535-023-02028-0.

## Introduction

Intraductal papillary mucinous neoplasm (IPMN) of the pancreas is a papillary lesion arising from the pancreatic epithelium, which potentially progresses to pancreatic cancer [1–4]. Accumulating evidence suggests that patients with IPMNs are also at high risk of developing concomitant pancreatic ductal adenocarcinoma (PDAC) [5–8]. Given the largely indolent biological behavior of the IPMNs, most patients diagnosed with IPMNs are subjected to the surveillance based on abdominal imaging studies [5, 6, 8–11]. However, the surveilled patients are occasionally diagnosed with pancreatic cancer at advanced stage when the disease is not curable [6, 12]. The prognosis of patients with advanced pancreatic cancer has been dismal [13], and therefore, it is mandatory to clarify alterations in serological and morphological characteristics of the IPMNs during the carcinogenic process to design effective surveillance programs for early diagnosis.

Clinical studies point to the potentials of serum carbohydrate antigen 19-9 (CA19-9) and hemoglobin A1c (HbA1c) as biomarkers for early detection of PDAC in general populations [14–17]. The levels of CA19-9 may exhibit an abrupt increase approximately 6–24 months before clinical manifestation of pancreatic cancer [14]. Compared to individuals with no diabetes mellitus, individuals with long-standing diabetes may be at approximately 1.4-fold elevated risk of pancreatic cancer [16]. In addition, new-onset diabetes may be a diagnostic clue for pancreatic cancer [18–22]. A fraction of patients diagnosed with pancreatic cancer develop acute pancreatitis before the cancer diagnosis due to the obstruction of the pancreatic duct [23]. Pancreatic carcinomas occurring concomitantly among patients with IPMNs share the common molecular pathological background with incidental PDACs to some extent [24]. In cross-sectional analyses of surgical series of IPMNs, aberrant CA19-9 elevation and diabetes were associated with high prevalence of concomitant PDAC in surgical specimens [17, 25]. However, no study has investigated the potential roles of CA19-9, HbA1c, and pancreatic enzymes in the context of long-term surveillance of patients with IPMNs for cancer screening.

Morphological features of IPMNs that are highly suggestive of pancreatic cancer development have been documented extensively by cross-sectional studies [26–28]. The international consensus guideline has proposed “worrisome features” and “high-risk stigmata” as predictive factors for the existence of carcinoma lesions in surgical specimens of IPMNs [29]. Due to the paucity of long-term follow-up data, however, the morphological progression of IPMNs before pancreatic carcinoma diagnosis has not been well characterized.

To characterize the alterations in the serological and morphological markers during pancreatic carcinogenesis among patients with IPMNs, we leveraged data on 100 patients who were diagnosed with pancreatic carcinomas within a large prospective cohort with follow-up duration of up to 25 years. We examined the timings of abnormal elevation of blood markers (i.e., CA19-9, HbA1c, and pancreatic enzymes) and those of occurrence of worrisome features or high-risk stigmata before clinical diagnosis of pancreatic carcinomas, overall and by the carcinoma types (IPMN-derived carcinoma vs. concomitant PDAC).

## Methods

### Study population

**Fig. 1:**
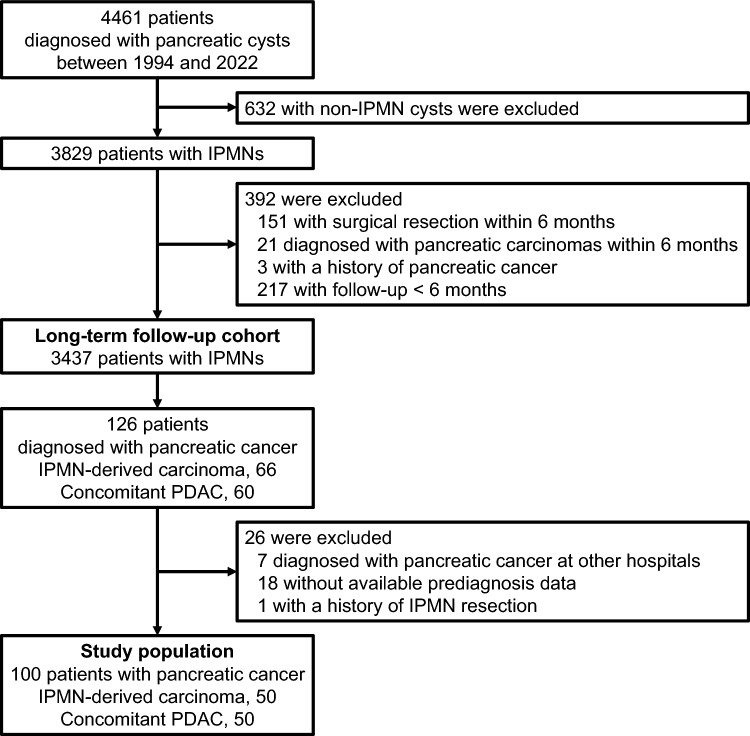
Flow diagram of selecting IPMN patients diagnosed with pancreatic carcinomas as the study population. *IPMN* intraductal papillary mucinous neoplasm, *PDAC* pancreatic ductal adenocarcinoma

In our prospectively maintained database, we have collected data on consecutive patients diagnosed with pancreatic cystic lesions including IPMNs at The University of Tokyo Hospital (Tokyo, Japan) [30, 31]. The diagnosis of IPMNs was made based on imaging findings according to the current consensus guideline proposed by the International Association of Pancreatology in 2017 [29]. We reevaluated the patients who had been diagnosed with IPMNs before the publication of the current version of the guideline. Branch-duct IPMNs were defined as unilocular or multilocular pancreatic cystic lesions that communicate with the main pancreatic duct (MPD), and main-duct IPMNs were defined as segmental or diffuse dilatation of the MPD of > 5 mm without other causes of the MPD dilatation. Mixed-type IPMNs were defined as lesions meeting the diagnostic criteria both for branch-duct and main-duct IPMNs. Among patients diagnosed with IPMNs from January 1994 through August 2022, we included pancreatic carcinoma patients with available prediagnosis information in the current study (Fig. 1). The patients were followed until death or the end of follow-up (September 30, 2022), whichever came first. At baseline of long-term follow-up, we excluded patients who underwent surgical resection of the IPMN or a diagnosis of pancreatic carcinoma within 6 months of the IPMN diagnosis, had a history of pancreatic carcinoma, or had follow-up time of < 6 months. In analyses of the trajectory of CA19-9 levels, we excluded the cases where data within 1 year preceding pancreatic carcinoma diagnosis were unavailable. As sensitivity analyses, we further excluded plausible Lewis antigen-negative cases with CA19-9 levels below the detection sensitivity (*n* = 8) [32] and confirmed that our findings did not change materially (data not shown).

This study was conducted according to the guidelines in the Helsinki Declaration and was approved by the ethics committee of The University of Tokyo (Tokyo, Japan; #1804, 2058, and G0500). Informed consent was obtained from the participants on an opt-out basis given the non-invasive nature of the study.

### Surveillance of IPMNs and ascertainment of pancreatic carcinoma cases

**Fig. 2:**
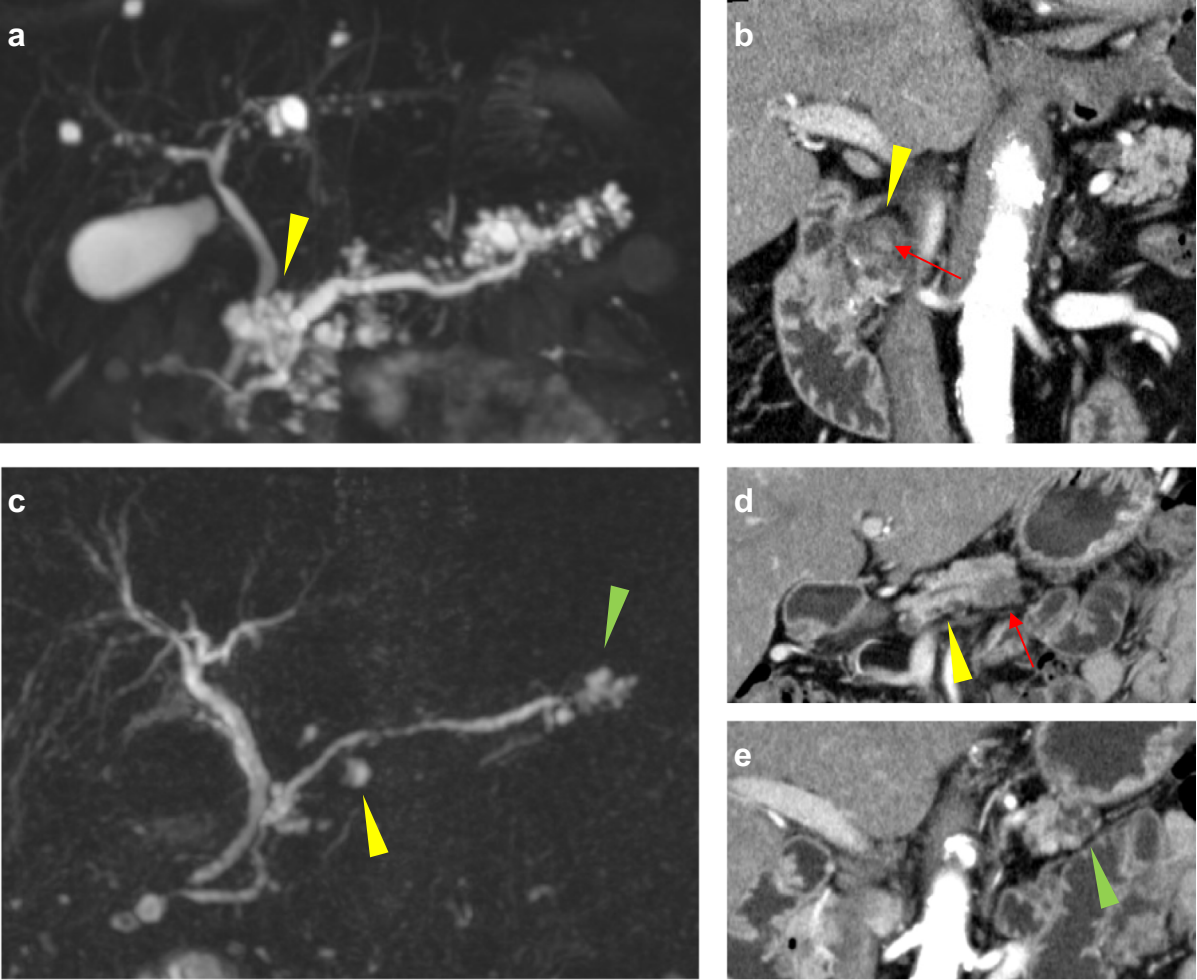
Patients diagnosed with pancreatic carcinomas during long-term surveillance of IPMNs. **a** and **b** Case with an IPMN-derived carcinoma. MRCP showed no mural nodule at the diagnosis of IPMNs at the whole pancreas (**a**). After follow-up of 7.8 years, contrast-enhanced CT showed a mural nodule in the pre-existing IPMN at the pancreatic head (**b**). The arrowhead in each figure indicates an IPMN, and the arrow indicates an enhancing mural nodule. **c**–**e** Case with a concomitant PDAC. Baseline MRCP showed several IPMN lesions at the body to tail of the pancreas without worrisome features or high-risk stigmata (**c**). After follow-up of 3.6 years, contrast-enhanced CT revealed a concomitant PDAC at the body of the pancreas (**d** and **e**). The arrowhead in each figure indicates an IPMN, and the arrow indicates a hypovascular mass suggestive of a concomitant PDAC. *CT* computed tomography, *IPMN* intraductal papillary mucinous neoplasm, *MRCP* magnetic resonance cholangiopancreatography, *PDAC* pancreatic ductal adenocarcinoma

The patients visited our outpatient clinic every 6 months and underwent physical examinations along with blood tests including levels of CA19-9, CEACAM5 (carcinoembryonic antigen [CEA]), HbA1c, and amylase (plus pancreatic amylase and lipase measured on the physicians’ discretion). At the same interval, we also performed imaging tests including magnetic resonance imaging (MRI) along with magnetic resonance cholangiopancreatography and others (abdominal ultrasound [AUS], endoscopic ultrasound, and contrast-enhanced computed tomography [CT]). The patients have undergone MRI at least once a year, regardless of the characteristics of IPMNs. Therefore, our surveillance has been more intense than the recommendation by the international consensus guideline [29]. When any signs suggesting pancreatic carcinoma development were demonstrated on imaging modalities during the follow-up period, we performed endoscopic ultrasound-guided fine-needle aspiration (EUS-FNA) and/or endoscopic retrograde cholangiopancreatography (ERCP) to confirm a cytological or histopathological diagnosis of pancreatic carcinoma [33]. According to the local consensus in Japan [29], EUS-FNA was not performed for the analysis and cytology of cyst fluid. For resected cases, the final diagnosis was made based on pathological examinations of surgical specimens. When tissue specimens were not available, the diagnosis of pancreatic carcinoma was made based on typical radiological findings with compatible clinical course. Invasive carcinoma and IPMN with high-grade dysplasia (formerly referred to as intraductal papillary mucinous carcinoma) were considered as malignant [34] and analyzed as IPMN-derived carcinoma. IPMN-derived carcinoma and concomitant PDAC were differentiated based on the radiological and/or pathological assessments of the continuity of carcinoma and IPMN (Fig. 2).

### Assessment of CA19-9, HbA1c, and pancreatic enzymes before pancreatic carcinoma diagnosis

In analyses of CA19-9 levels, we defined the time-points of twofold, threefold, and fivefold increases compared to the case-specific reference value for graphical presentations. We categorized trajectory patterns of CA19-9 into the following groups: aberrant elevation, patients with the first documentation of the CA19-9 level above the upper limit of normal (> 37 U/mL) [10] or doubling of the cumulative average (above the upper limit of normal), within 1 year preceding pancreatic carcinoma diagnosis; low to low, patients with CA19-9 levels within normal throughout the follow-up period before pancreatic carcinoma diagnosis; and high to high, patients with elevated values of CA19-9 at baseline and without doubling before pancreatic carcinoma diagnosis. We defined HbA1c elevation when there was a 4 mmol/mol increase compared to the cumulative average [22]. We defined aberrant elevation of a pancreatic enzyme when pancreatic amylase or lipase was above triple the upper limit of normal with the doubled value of the cumulative average within 1 year preceding pancreatic carcinoma diagnosis. We used the cumulative averages as the reference values considering intra-individual variations of the markers.

### Assessment of morphologic features of IPMNs before pancreatic carcinoma diagnosis

We characterized morphologic features of IPMNs before pancreatic carcinoma diagnosis focusing on worrisome features and high-risk stigmata, which had been defined according to the current international consensus guideline [29]. The guideline recommends surgical resection for IPMNs harboring high-risk stigmata and a further examination via endoscopic ultrasound for IPMNs harboring worrisome features. The high-risk stigmata include (1) obstructive jaundice in a patient with an IPMN at the pancreatic head, (2) an enhanced mural nodule ≥ 5 mm, and (3) the MPD diameter of ≥ 10 mm. The worrisome features include (1) the IPMN size of ≥ 30 mm, (2) an enhancing mural nodule < 5 mm, (3) thickened enhanced wall, (4) the MPD diameter of 5–9.9 mm, ((5) an abrupt caliber change of the MPD with distal pancreatic atrophy, (6) lymphadenopathy, (7) an elevated serum level of CA19-9 (> 37 U/mL), (8) a growth rate of > 5 mm/2 years, and (9) acute pancreatitis. We reviewed all imaging and laboratory tests before pancreatic carcinoma diagnosis and recorded the time-point of the first documentation of worrisome features or high-risk stigmata.

### Statistical analysis

Standard descriptive statistics and graphical presentations were used to document the trajectory of serological and morphological markers before pancreatic carcinoma diagnosis. Continuous variables were compared using the Wilcoxon rank-sum test, and categorical variables were compared using the Chi-square test or Fisher’s exact test, as appropriate. In survival analyses, we examined associations between prediagnosis CA19-9 trajectory patterns and overall survival among patients diagnosed with pancreatic cancer, stratified by the carcinoma types. Overall survival time was defined as the time from the diagnosis of carcinoma to death of any cause or the end of follow-up, where patients who were alive at the last follow-up were censored. Cumulative survival probabilities were estimated using the Kaplan–Meier product-limit method and were compared using the log-rank test.

Statistical analyses were performed using R software (version 3.5.1, R Development Core Team). Two-sided *P* values < 0.05 were considered statistically significant.

## Results

### Clinical characteristics at IPMN diagnosis and pancreatic carcinoma diagnosis

Within our clinical cohort, we have followed up 4461 patients with pancreatic cysts, including 3437 IPMN patients with long-term surveillance. We documented 100 pancreatic carcinoma cases during 19,795 person-years of follow-up of the IPMN patients with median duration of 5.7 years (range 0.5–25.6 years) (Fig. 1). Table 1 and Supplementary Table 1 summarize the characteristics of the 100 patients (IPMN-derived and concomitant carcinomas each in 50 patients) at pancreatic carcinoma diagnosis and IPMN diagnosis, respectively. At baseline, there were 58 males and 42 females with a mean age of 70.9 years (range 43–88 years). The IPMNs at baseline were branch-duct type, main-duct type, and mixed type in 80, 6, and 14 patients, respectively, and harbored worrisome features and high-risk stigmata in 23 and 5 patients, respectively. At the time of pancreatic carcinoma diagnosis, diabetes mellitus was present in 49 patients including 9 patients with recent-onset (within 2 years) diabetes, and CA19-9 elevation was observed in 63 patients including 39 patients with aberrant elevation. Diagnostic modalities for pancreatic carcinomas and postdiagnostic treatment are summarized in Supplementary Table 2.

**Table 1:** Characteristics of patients diagnosed with pancreatic carcinomas during long-term surveillance of intraductal papillary mucinous neoplasms at pancreatic carcinoma diagnosis, overall and by carcinoma types

Characteristic^a^	All cases (*n* = 100)	Carcinoma type		*P* value
		IPMN-derived (*n* = 50)	Concomitant (*n* = 50)	
Age, years	75.9 ± 8.0	76.0 ± 9.0	75.7 ± 7.0	0.85
Sex				0.69
Male	58 (58%)	30 (60%)	28 (56%)	
Female	42 (42%)	20 (40%)	22 (44%)	
Symptom				0.28
Absent	84 (84%)	44 (88%)	40 (80%)	
Present	16 (16%)	6 (12%)	10 (20%)	
Acute pancreatitis				0.65
Absent	95 (95%)	48 (96%)	47 (94%)	
Present	5 (5%)	2 (4%)	3 (6%)	
Smoking status				0.55
Never	47 (47%)	22 (44%)	25 (50%)	
Past/current	53 (53%)	28 (56%)	25 (50%)	
Body mass index, kg/m^2^	21.9 ± 3.3	22.0 ± 3.6	21.9 ± 3.1	0.90
Diabetes mellitus				0.66
Absent	51 (51%)	25 (50%)	26 (52%)	
Recent onset (< 2 years)	9 (9%)	6 (12%)	3 (6%)	
Unknown onset	3 (3%)	2 (4%)	1 (2%)	
Long-standing (≥ 2 years)	37 (37%)	17 (34%)	20 (40%)	
Family history of pancreatic cancer				0.24
Absent	93 (93%)	48 (96%)	45 (90%)	
Present	7 (7%)	2 (4%)	5 (10%)	
Amylase, U/L	87 (14–2897)	104 (14–2897)	84 (20–805)	0.61
Pancreatic amylase, U/L	30 (5–2813)	31 (6–2813)	27.5 (5–695)	0.99
Lipase, U/L	45 (9–1406)	44 (9–1182)	48.5 (10–1406)	0.31
HbA1c, %	6.4 (5.0–10.2)	6.2 (5.0–10.2)	6.7 (5.4–10.1)	0.04
CA19-9, U/mL	65 (1–21,970)	43.5 (1–1886)	177 (1–21,970)	0.002
CEACAM5 (CEA), ng/mL	4.7 (0.9–237.4)	4.7 (0.9–81.5)	4.8 (1.5–237.4)	0.34
Location of carcinoma				0.55
Head	51 (51%)	27 (54%)	24 (48%)	
Body-tail	49 (49%)	23 (46%)	26 (52%)	
Diameter of the MPD				0.03
< 5 mm	45 (45%)	16 (32%)	29 (58%)	
5–9.9 mm	35 (35%)	21 (42%)	14 (28%)	
≥ 10 mm	20 (20%)	13 (26%)	7 (14%)	
Clinical cancer stage^b^				< 0.001
0	19 (19%)	18 (36%)	1 (2%)	
I	18 (18%)	10 (20%)	8 (16%)	
II	42 (42%)	15 (30%)	27 (54%)	
T3N0M0	35	12	23	
T3N1M0	7	3	4	
III	11 (11%)	5 (10%)	6 (12%)	
T4N0M0	8	4	4	
T4N1M0	2	1	1	
T2N2M0	1	0	1	
IV	10 (10%)	2 (4%)	8 (16%)	
Pathological cancer stage^b^				< 0.001
0	17 (25%)	16 (43%)	1 (3.2%)	
I	6 (8.8%)	5 (14%)	1 (3.2%)	
II	39 (57%)	14 (38%)	25 (81%)	
T3N0M0	21	9	12	
T3N1M0	16	4	11	
T2N1M0	2	0	2	
T1N1M0	1	1	0	
III	3 (4.4%)	1 (2.7%)	2 (6.5%)	
T3N2M0	2	1	1	
T4N0M0	1	0	1	
IV	3 (4.4%)	1 (2.7%)	2 (6.5%)	

*CA19-9* carbohydrate antigen 19-9, *CEA* carcinoembryonic antigen, *HbA1c* hemoglobin A1c, *IPMN* intraductal papillary mucinous neoplasm, *MPD* main pancreatic duct

^a^Data are presented as mean ± standard deviation, median (range), or number of patients (%). Percentage indicates the proportion of patients with a specific characteristic in all cases or strata of the carcinoma types. Total percentages may not equal 100% due to rounding

^b^Five categories of cancer stage (0 vs. I vs. II vs. III vs. IV) were compared

### Serological and morphological abnormalities at the time of pancreatic carcinoma diagnosis

Table 2 presents pancreatic carcinoma subgroups jointly defined by the abnormalities in laboratory and imaging tests at the time of pancreatic carcinoma diagnosis. Elevated levels of CA19-9, HbA1c, and pancreatic enzymes were observed in 39, 3, and 18 patients, respectively, whereas all items remained with no aberrant elevation in 48 patients. In 82 patients, follow-up imaging studies delineated abnormal findings suggesting pancreatic carcinoma development (e.g., development or progression of a mural nodule, development of a solid mass). For all 10 patients without abnormal imaging findings, the modality performed was abdominal ultrasound, and 9 (90%) of those patients had elevated CA19-9 levels, which prompted a further evaluation and resulted in pancreatic carcinoma diagnosis immediately thereafter. Given the quite limited number of cases with HbA1c elevation, we did not examine prediagnostic trajectory of this marker.

**Table 2:** Abnormal findings on laboratory tests and imaging studies at the time of diagnosis of pancreatic carcinomas occurring among patients with intraductal papillary mucinous neoplasms

	Abnormal laboratory results^a^					
	Present (*n* = 52)					Absent (*n* = 48)
	CA19-9 and pancreatic enzyme (*n* = 6)	CA19-9 and HbA1c (*n* = 2)	CA19-9 (*n* = 31)	Pancreatic enzyme (*n* = 12)	HbA1c (*n* = 1)	
Abnormal imaging findings						
Present						
AUS (*n* = 26)	3	1	5	4		13
CT (*n* = 19)	1		4	3		11
MRI/MRCP (*n* = 22)			5	1	1	15
EUS (*n* = 15)	1	1	6	2		5
Absent						
AUS (*n* = 10)^b^			9			1
No imaging (*n* = 8)	1		2	2		3

*AUS* abdominal ultrasound, *CA19-9* carbohydrate antigen 19-9, *CT* computed tomography, *EUS* endoscopic ultrasound, *HbA1c* hemoglobin A1c, *MRCP* magnetic resonance cholangiopancreatography, *MRI* magnetic resonance imaging

^a^We defined aberrant CA19-9 elevation for patients with the first documentation of the CA19-9 level above the upper limit of normal (> 37 U/mL) or doubling of the cumulative average (above the upper limit of normal), within 1 year preceding pancreatic carcinoma diagnosis. We defined aberrant elevation of a pancreatic enzyme when pancreatic amylase or lipase was above 3 times the upper limit of normal with the doubled value of the cumulative average within 1 year preceding pancreatic carcinoma diagnosis. We defined aberrant HbA1c elevation when there was a 4 mmol/mol increase compared to the cumulative average within 1 year preceding pancreatic carcinoma diagnosis

^b^For all 10 patients, pancreatic carcinoma was detected by contrast-enhanced CT immediately following the failed detection by AUS

### Trajectories of CA19-9 levels and pancreatic enzymes before pancreatic carcinoma diagnosis

**Fig. 3:**
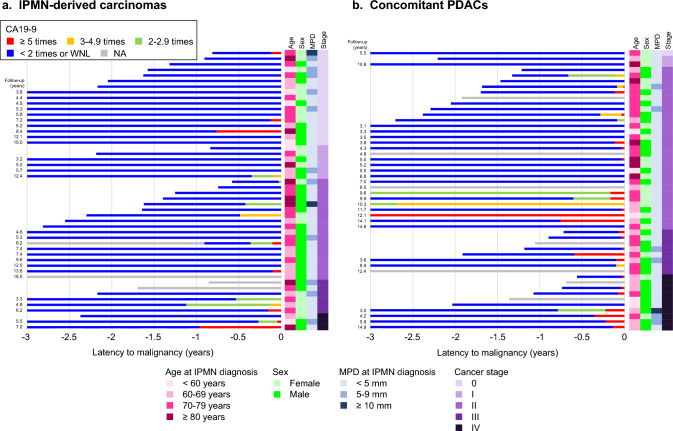
Trajectory of serum CA19-9 levels before pancreatic carcinoma diagnosis among patients with IPMNs, by carcinoma types and stages. **a** IPMN-derived carcinomas (*n* = 50) and **b** concomitant PDACs (*n* = 50). *CA19-9* carbohydrate antigen 19-9, *IPMN* intraductal papillary mucinous neoplasm, *MPD* main pancreatic duct, *NA* not available, *PDAC* pancreatic ductal adenocarcinoma, *WNL* within normal limit

Figure 3 illustrates longitudinal changes of CA19-9 levels among patients diagnosed with IPMN-derived and concomitant carcinomas according to cancer stage (the graphs sorted by follow-up duration illustrated in Supplementary Fig. 1 and raw data on CA19-9 levels graphically presented in Supplementary Fig. 2). Compared to patients with IPMN-derived carcinomas, patients with concomitant PDACs were more likely to have elevated CA19-9 levels (60% vs. 30%, respectively; *P* = 0.005). The time from CA19-9 elevation to pancreatic carcinoma diagnosis did not differ by the carcinoma types: 0.4 (interquartile range [IQR] 0.1–0.5) years in IPMN-derived carcinomas and 0.1 (IQR 0.1–0.7) years in concomitant PDACs (*P* = 0.23). Figure 3 additionally demonstrates a lower likelihood of CA19-9 elevation at earlier stages of both carcinoma types (23% vs. 71% in stage 0–II and III–IV of IPMN-derived carcinomas, respectively [*P* = 0.009]; and 41% vs. 100% in stage 0–II and III–IV of concomitant PDACs, respectively [*P* < 0.001]). Table 3 presents the characteristics of pancreatic carcinomas by the trajectory patterns of CA19-9 levels. The *aberrant elevation* group was characterized by a high proportion of concomitant PDAC (*P* = 0.003) and advanced stage (*P* < 0.001). In an analysis of postdiagnosis survival times (Supplementary Fig. 3), pancreatic carcinomas with aberrant elevation of CA19-9 were associated with high mortality compared to carcinomas with no aberrant elevation.

**Table 3:** Carcinoma types and stages at the diagnosis of pancreatic carcinomas according to CA19-9 trajectory patterns during long-term surveillance of intraductal papillary mucinous neoplasms

Carcinoma characteristic^b^	CA19-9 trajectory pattern^a^			*P* value
	Aberrant elevation (*n* = 39)	High to high (*n* = 12)	Low to low (*n* = 38)	
Type				0.003
IPMN-derived carcinoma (*n* = 47)	14 (30%)	10 (21%)	23 (49%)	
Concomitant PDAC (*n* = 42)	25 (60%)	2 (4.8%)	15 (36%)	
Stage				< 0.001
0/I (*n* = 26)	3 (12%)	8 (31%)	15 (58%)	
II (*n* = 43)	18 (42%)	3 (7.0%)	22 (51%)	
III (*n* = 10)	9 (90%)	1 (10%)	0	
IV (*n* = 10)	9 (90%)	0	1 (10%)	

*CA19-9* carbohydrate antigen 19-9, *IPMN* intraductal papillary mucinous neoplasm, *PDAC* pancreatic ductal adenocarcinoma

^a^We categorized trajectory patterns of CA19-9 into the following groups: aberrant elevation, patients with the first documentation of the CA19-9 level above the upper limit of normal (> 37 U/mL) or doubling of the cumulative average (above the upper limit of normal), within 1 year preceding pancreatic carcinoma diagnosis; low to low, patients with CA19-9 levels within normal throughout the follow-up period before pancreatic carcinoma diagnosis; and high to high, patients with elevated values of CA19-9 at baseline and without doubling before pancreatic carcinoma diagnosis. Patients without available data on CA19-9 within 1 year preceding pancreatic carcinoma diagnosis (*n* = 11) were excluded from the analysis

^b^Data are presented as number of patients (%). Percentage indicates the proportion of patients with a specific CA19-9 trajectory pattern in strata of carcinoma characteristics. Total percentages may not equal 100% due to rounding

Acute pancreatitis developed before the carcinoma diagnosis only in five patients. Supplementary Fig. 4 graphically presents the trajectory of pancreatic enzymes and occurrence of acute pancreatitis before pancreatic carcinoma diagnosis among patients with IPMNs. Aberrant elevation of any pancreatic enzymes was observed in 18 patients with no statistically significant difference between the carcinoma types (16% vs. 20% in IPMN-derived and concomitant carcinomas, respectively; *P* = 0.60). In addition, there was no statistically significant difference in latency times to carcinoma diagnosis (4.5 years [IQR 1.5–10.2 years] vs. 1.6 years [IQR 1.2–3.7 years] for IPMN-derived and concomitant carcinomas, respectively; *P* = 0.52). However, the small number of cases with the aberrant elevation precluded a robust statistical assessment.

### Trajectory of morphological features before pancreatic carcinoma diagnosis

**Fig. 4:**
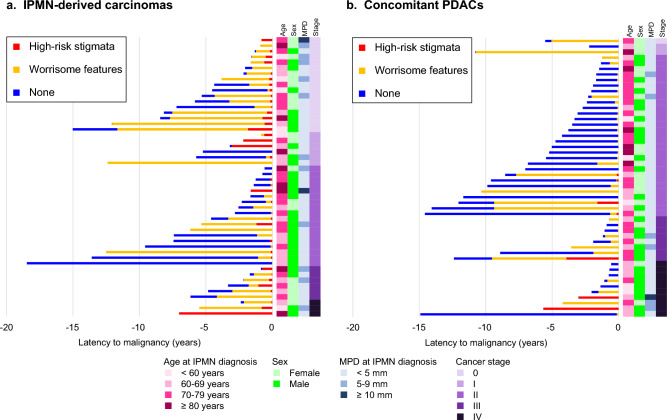
Trajectory of morphologic features of IPMNs before pancreatic carcinoma diagnosis among patients with IPMNs, by carcinoma types and stages. **a** IPMN-derived carcinomas (*n* = 50) and **b** concomitant PDACs (*n* = 50). *IPMN* intraductal papillary mucinous neoplasm, *MPD* main pancreatic duct, *PDAC* pancreatic ductal adenocarcinoma

Figure 4 illustrates longitudinal changes of morphologic features of IPMNs among patients diagnosed with IPMN-derived and concomitant carcinomas according to cancer stage (the graphs sorted by follow-up duration illustrated in Supplementary Fig. 5). The types of worrisome features and high-risk stigmata observed during the long-term surveillance and the timing of their occurrence are summarized in Supplementary Table 3 and Supplementary Fig. 6. The types of worrisome features and high-risk stigmata that were eventually observed at carcinoma diagnosis are summarized in Supplementary Table 4. At least one of worrisome features or high-risk stigmata developed in all patients but one with a concomitant PDAC. Compared to IPMN-derived carcinomas, concomitant PDACs were less likely to represent high-risk stigmata (16% vs. 86%, respectively; *P* < 0.001). As graphically presented in Fig. 4, worrisome features and high-risk stigmata were less likely observed before the clinical manifestation of stage 0-II concomitant PDACs. The time from the documentation of worrisome features or high-risk stigmata to pancreatic carcinoma diagnosis was significantly shorter in concomitant PDACs compared to IPMN-derived carcinomas (0.2 [IQR 0.1–1.9] years and 1.6 [IQR 0.7–3.7] years, respectively; *P* = 0.002).

## Discussion

In this large prospective series of pancreatic carcinoma cases identified during long-term follow-up of IPMNs, we characterized the temporal changes of serological and morphological features prior to the development of pancreatic carcinomas and examined the heterogeneity in those alterations between the carcinoma types. Aberrant elevation of serum CA19-9 levels was observed in up to 60% patients developing concomitant PDACs but less frequently in patients developing IPMN-derived carcinomas. In addition, the carcinomas with the CA19-9 elevation were detected reliably by contrast-enhanced CT or MRI, casting doubt on the effectiveness of the surveillance based on CA19-9. The levels of HbA1c rarely elevated before the pancreatic carcinogenesis, irrespective of the carcinoma types. Aberrant elevation of pancreatic enzymes was observed in 18% patients, but clinically evident pancreatitis developed only in 5% patients. The current study also demonstrated distinctive patterns of morphological alterations for respective carcinoma types occurring among patients with IPMNs (IPMN-derived vs. concomitant carcinomas). Concomitant PDACs frequently arise without high-risk stigmata characteristic of IPMN-derived carcinomas. These findings underscore the importance of developing more sensitive biomarkers and imaging protocols to detect early-stage pancreatic neoplasms in patients with IPMNs.

The current study points to the limited potentials of the blood biomarkers that have been investigated in the setting of early diagnosis of incidental PDAC. CA19-9 is one of the most promising biomarkers for PDAC development and has been widely utilized in patients with PDACs for postoperative surveillance and tumor burden monitoring under chemotherapy [35–39]. In a case–control study in the U.S. [14], an exponential increase in serum CA19-9 levels started to be observed at approximately 2 years before pancreatic cancer diagnosis, and the diagnostic abilities (estimated by the area under the curve) increased thereafter. Given these lines of evidence, CA19-9 has been incorporated into the criteria for surgical indications in the clinical guidelines of IPMNs [9, 10, 29, 40]. However, our study implicates the limited sensitivity and specificity of serum CA19-9 in the long-term cancer monitoring for patients with IPMNs. We noted abnormal imaging findings suggestive of pancreatic carcinoma development when aberrant CA19-9 elevation was observed, calling into question the surveillance based on serum CA19-9 for detecting pancreatic cancer at the preclinical stage when the tumor exhibits no mass lesion. Aberrant CA19-9 elevation was observed in 60% of patients with concomitant PDACs in contrast to 30% of patients with IPMN-derived carcinomas; however, the elevation was observed shortly before the cancer diagnosis, and the cancer stage was III or IV in 50% patients with concomitant PDACs. Morphological alterations suggestive of the development of early-stage pancreatic cancer have been well characterized (MPD dilatation, an abrupt caliber change in the MPD, etc. [41]). MRI with magnetic resonance cholangiopancreatography can visualize the overall architecture of the MPD and help identify early signs of concomitant PDAC development. Therefore, it is of considerable importance to prudently evaluate the findings of follow-up MRI. In addition, given the reported effectiveness of EUS in the detection of mural nodules arising from IPMNs [42–44], EUS should be incorporated into surveillance programs for patients with MPD dilatation who have been at high risk of developing pancreatic carcinoma [45]. Our study also raised a concern on the specificity of CA19-9 by demonstrating that a fraction of patients with CA19-9 elevation remained free from developing pancreatic carcinomas (as shown in the high-to-high group). In addition, serum CA19-9 levels may be elevated under the presence of other adenocarcinomas such as colorectal and gastric cancer as well as benign cholestatic diseases [46, 47]. In the current study population, we did not perform cyst fluid analysis via EUS-FNA, which permits not only pathological examinations but also molecular profiling based on genomic annotations [48] and specific molecules (e.g., CEACAM5 [CEA], glucose). Several studies questioned the effectiveness of serial EUS-FNA for IPMNs [49, 50]; however, the highly sensitive sequencing technology opened opportunities for DNA-based screening based on circulating tumor DNA in pancreatic cancer (so-called liquid biopsy) [51, 52]. Prospective cohort studies are warranted to examine the integration of these new modalities into the current surveillance programs of IPMNs.

Our long-term analysis of IPMNs demonstrated distinctive morphological trajectories according to the carcinoma types by focusing on the timing of occurrence of worrisome features and high-risk stigmata during the carcinogenic process. Of note, worrisome features were observed in the vast majority of carcinomas irrespective of the histological types, but the repertoire of worrisome features differed between the carcinoma types. As expected, most IPMN-derived carcinomas represented morphological worrisome features before the carcinoma diagnosis (i.e., the MPD dilatation and the large cyst size). Most concomitant PDACs became positive for worrisome features by increasing CA19-9 levels immediately before the diagnosis and represented no high-risk stigmata. In addition, our previous study has shown that the long-term risk of concomitant PDACs may not be stratified based on the size of IPMN, the MPD diameter, or the presence of a mural nodule at the IPMN diagnosis [6]. These findings do not support morphology-based stratification of IPMN patients in terms of the long-term risk of developing concomitant PDAC. Given the substantial proportion of concomitant PDACs in carcinomas occurring among IPMN patients, there is a great need for a reliable analytical platform of imaging studies. The emerging artificial intelligence-based technology (e.g., radiomics and the convolutional neural network) may provide a promising approach for early radiological detection of concomitant PDACs [53–56], and the application of such technology is warranted in the setting of the IPMN surveillance.

The current study has notable strengths. To our knowledge, our study included the largest number of patients who developed pancreatic carcinomas during long-term surveillance of IPMNs. With the large sample size, we successfully characterized the differential clinical courses of IPMN-related pancreatic carcinomas according to the carcinoma types. Furthermore, the long follow-up duration of up to 25 years allowed us to map the starting points of the serological and morphological abnormalities on the long course of the carcinogenesis among patients with IPMNs.

We should acknowledge several limitations in our study. First, the dataset was derived from patients diagnosed with IPMNs at a single tertiary referral center, potentially resulting in a selection bias. Nonetheless, the prospective inclusion of consecutive patients with IPMNs minimized a selection bias within our institution and increased the generalizability of our findings. Second, there were variations in the timing and interval of blood tests and imaging studies between the patients. In indolent tumors requiring long duration until the development of symptoms, the time from the last surveillance to the clinical carcinoma diagnosis might depend on the interval between the surveillance examinations. Finally, a vast majority of the study population was Japanese, and therefore, our findings should be validated in independent cohorts.

In conclusion, our long-term data implicate the limited ability of the currently available blood biomarkers to identify early-stage pancreatic carcinomas during long-term surveillance of patients with IPMNs, particularly for IPMN-derived carcinomas. In addition, the diagnostic approach based on the distinctive morphological patterns of IPMN-derived carcinomas might not be feasible in detection of concomitant PDACs. Further research is warranted to develop multidisciplinary surveillance strategies integrated with the emerging technologies of liquid-based biomarker engineering and the imaging analysis.

### Supplementary Information

Below is the link to the electronic supplementary material.

Supplementary file1 (DOCX 1072 KB)

## Abbreviations

AUS: Abdominal ultrasound
CA19-9: Carbohydrate antigen 19-9
CEA: Carcinoembryonic antigen
CT: Computed tomography
ERCP: Endoscopic retrograde cholangiopancreatography
EUS-FNA: Endoscopic ultrasound-guided fine-needle aspiration
HbA1c: Hemoglobin A1c
IPMN: Intraductal papillary mucinous neoplasm
IQR: Interquartile range
MPD: Main pancreatic duct
MRI: Magnetic resonance imaging
PDAC: Pancreatic ductal adenocarcinoma
