# Supplementary material for: Clinical trajectory of intraductal papillary mucinous neoplasms progressing to pancreatic carcinomas during long-term surveillance: a prospective series of 100 carcinoma cases
Source: J Gastroenterol. 2023 Jul 29;58(10):1068–80. doi: 10.1007/s00535-023-02028-0 (PMC10522754; doi:10.1007/s00535-023-02028-0)
Supplement: Supplementary file 1 — Supplementary file1 (DOCX 1072 KB) [file 535_2023_2028_MOESM1_ESM.docx]

**Supplementary Information**

**Article title**

Clinical trajectory of intraductal papillary mucinous neoplasms progressing to pancreatic carcinomas during long-term surveillance: a prospective series of 100 carcinoma cases

**Journal name**

Journal of Gastroenterology

**Author names and affiliations**

Hiroki Oyama^1^, Tsuyoshi Hamada^1,2^, Yousuke Nakai^1,3^, Mariko Tanaka^4^, Go Endo^1^,

Ryunosuke Hakuta^1^, Kota Ishida^1^, Kazunaga Ishigaki^1^, Sachiko Kanai^1,3^,

Kohei Kurihara^1^, Tomotaka Saito^1^, Tatsuya Sato^1^, Tatsunori Suzuki^1^, Yukari Suzuki^1^,

Shinya Takaoka^1^, Shuichi Tange^1^, Yurie Tokito^1^, Naminatsu Takahara^1^,

Tetsuo Ushiku^4^, Mitsuhiro Fujishiro^1^

1. Department of Gastroenterology, Graduate School of Medicine, The University of Tokyo, Tokyo, Japan
2. Department of Hepato-Biliary-Pancreatic Medicine, The Cancer Institute Hospital of Japanese Foundation for Cancer Research, Tokyo, Japan
3. Department of Endoscopy and Endoscopic Surgery, The University of Tokyo Hospital, Tokyo, Japan
4. Department of Pathology, Graduate School of Medicine, The University of Tokyo, Tokyo, Japan

**Corresponding author**

Yousuke Nakai, MD, PhD

Department of Endoscopy and Endoscopic Surgery,

The University of Tokyo Hospital

7-3-1 Hongo, Bunkyo City, Tokyo 113-8655, Japan

Tel: +81-3-3815-5411

Fax: +81-3-3814-0021

E-mail: ynakai-tky@umin.ac.jp


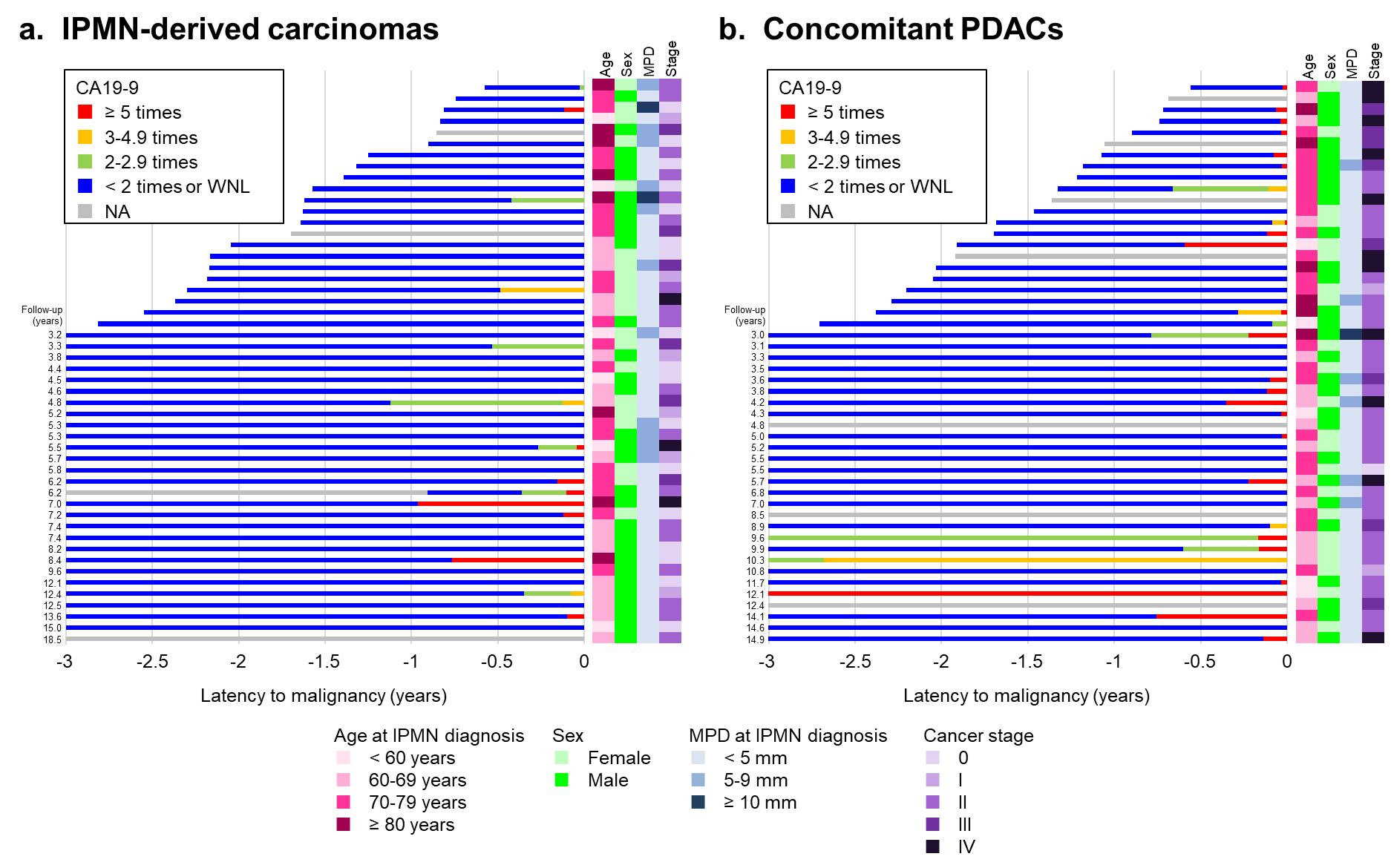


**Supplementary Fig. 1** Trajectory of serum CA19-9 levels before pancreatic carcinoma diagnosis among patients with IPMNs, by carcinoma types. (a) IPMN-derived carcinomas (*n* = 50) and (b) concomitant PDACs (*n* = 50).

Abbreviations: CA19-9, carbohydrate antigen 19-9; IPMN, intraductal papillary mucinous neoplasm; MPD, main pancreatic duct; NA, not available; PDAC, pancreatic ductal adenocarcinoma; WNL, within normal limit.


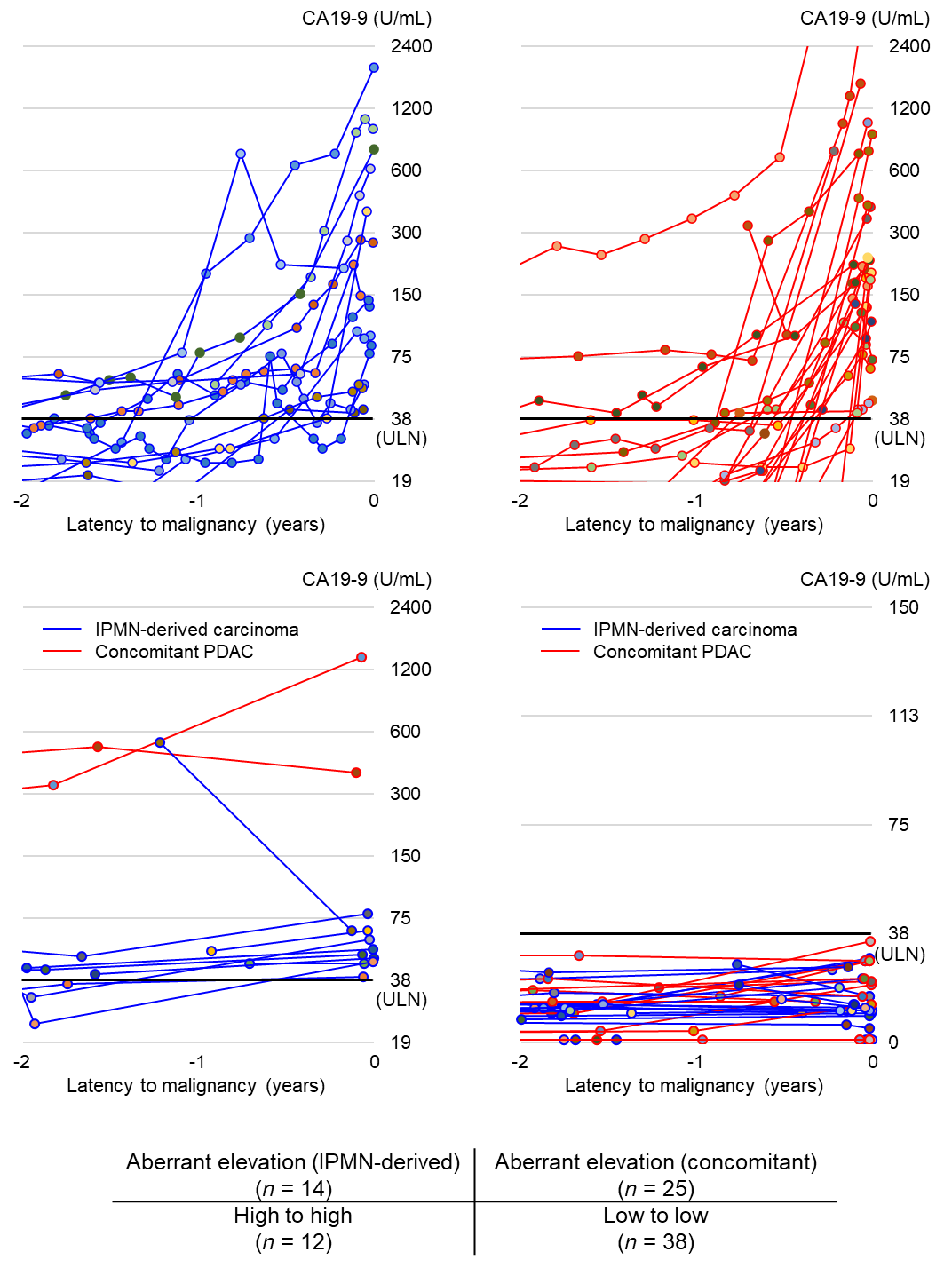


**Supplementary Fig. 2** Trajectory patterns of CA19-9 before the diagnosis of pancreatic carcinomas among patients with IPMNs under long-term surveillance.

Abbreviations: CA19-9, carbohydrate antigen 19-9; IPMN, intraductal papillary mucinous neoplasm; PDAC, pancreatic ductal adenocarcinoma; ULN, upper limit of normal.

**
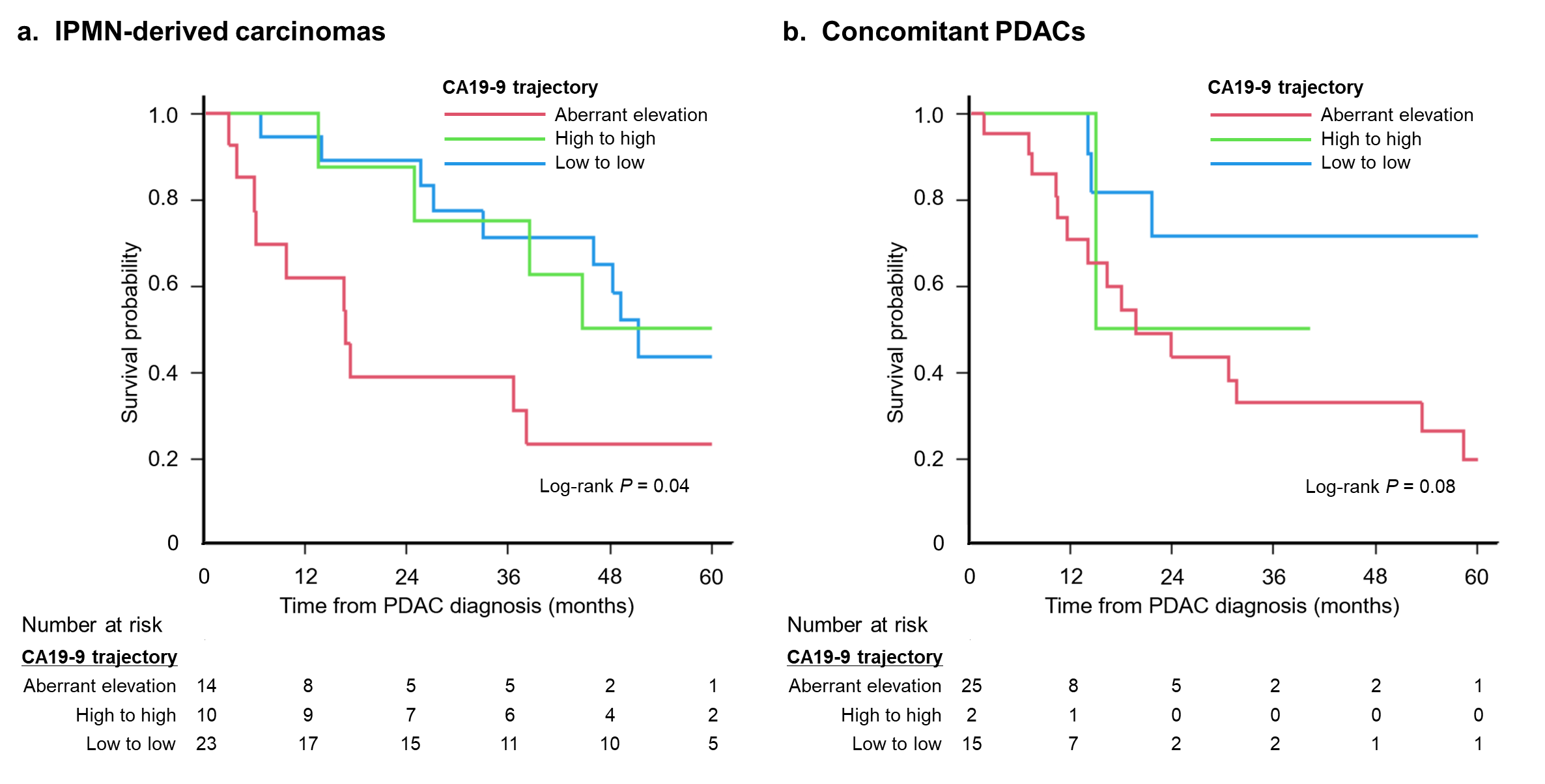
**

**Supplemenatary Fig. 3** Kaplan-Meier curves of overall survival among patients diagnosed with pancreatic carcinomas during long-term surveillance of IPMNs, by trajectory patterns of CA19-9. (a) IPMN-derived carcinomas (*n* = 50) and (b) concomitant PDACs (*n* = 50).

Abbreviations: CA19-9, carbohydrate antigen 19-9; IPMN, intraductal papillary mucinous neoplasm; PDAC, pancreatic ductal adenocarcinoma.
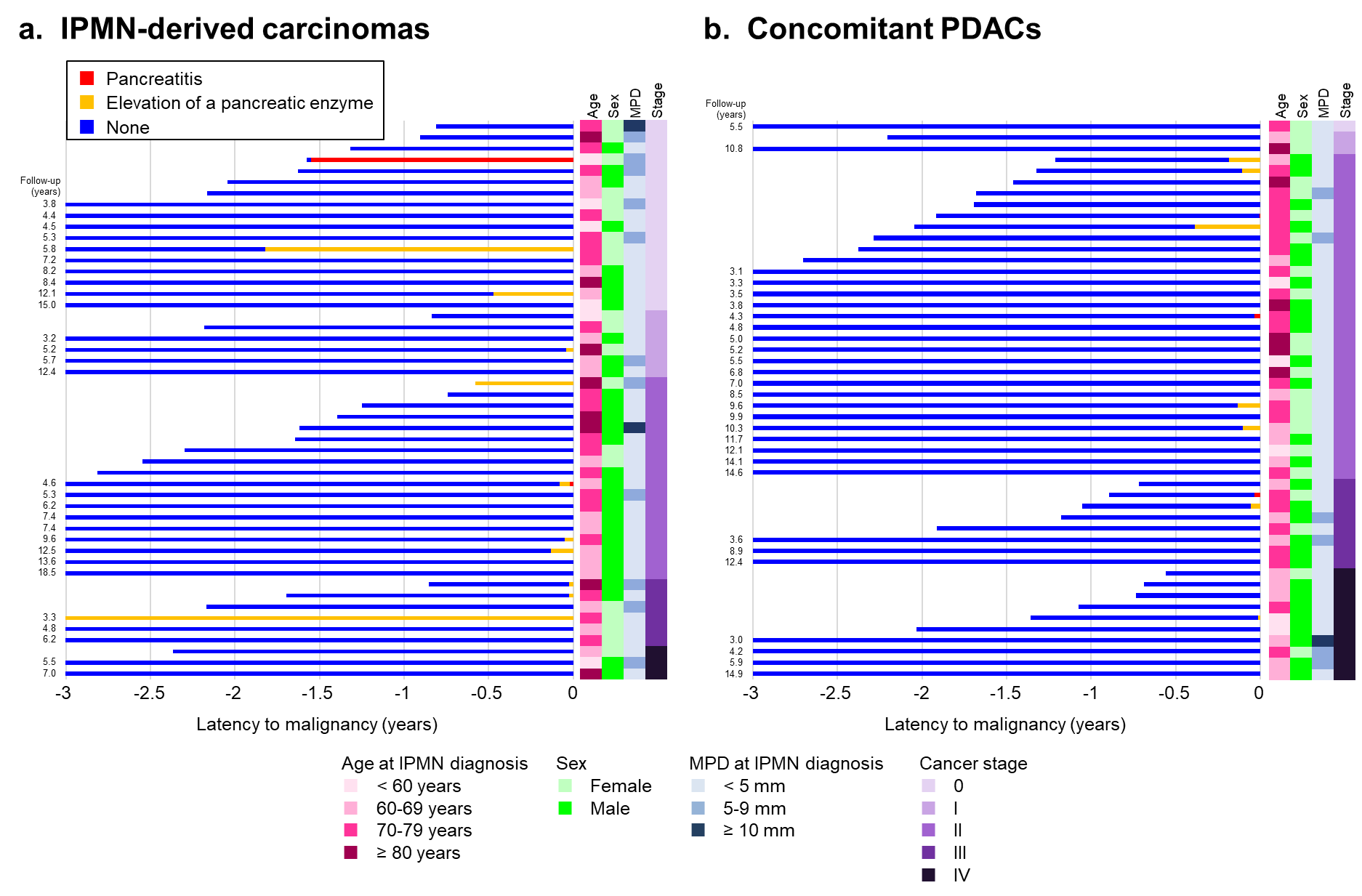


**Supplemenatary Fig. 4** Trajectory of pancreatic enzymes and occurrence of acute pancreatitis before pancreatic carcinoma diagnosis among patients with IPMNs, by carcinoma types. (a) IPMN-derived carcinomas (*n* = 50) and (b) concomitant PDACs (*n* = 50).

Abbreviations: IPMN, intraductal papillary mucinous neoplasm; MPD, main pancreatic duct; PDAC, pancreatic ductal adenocarcinoma.

**
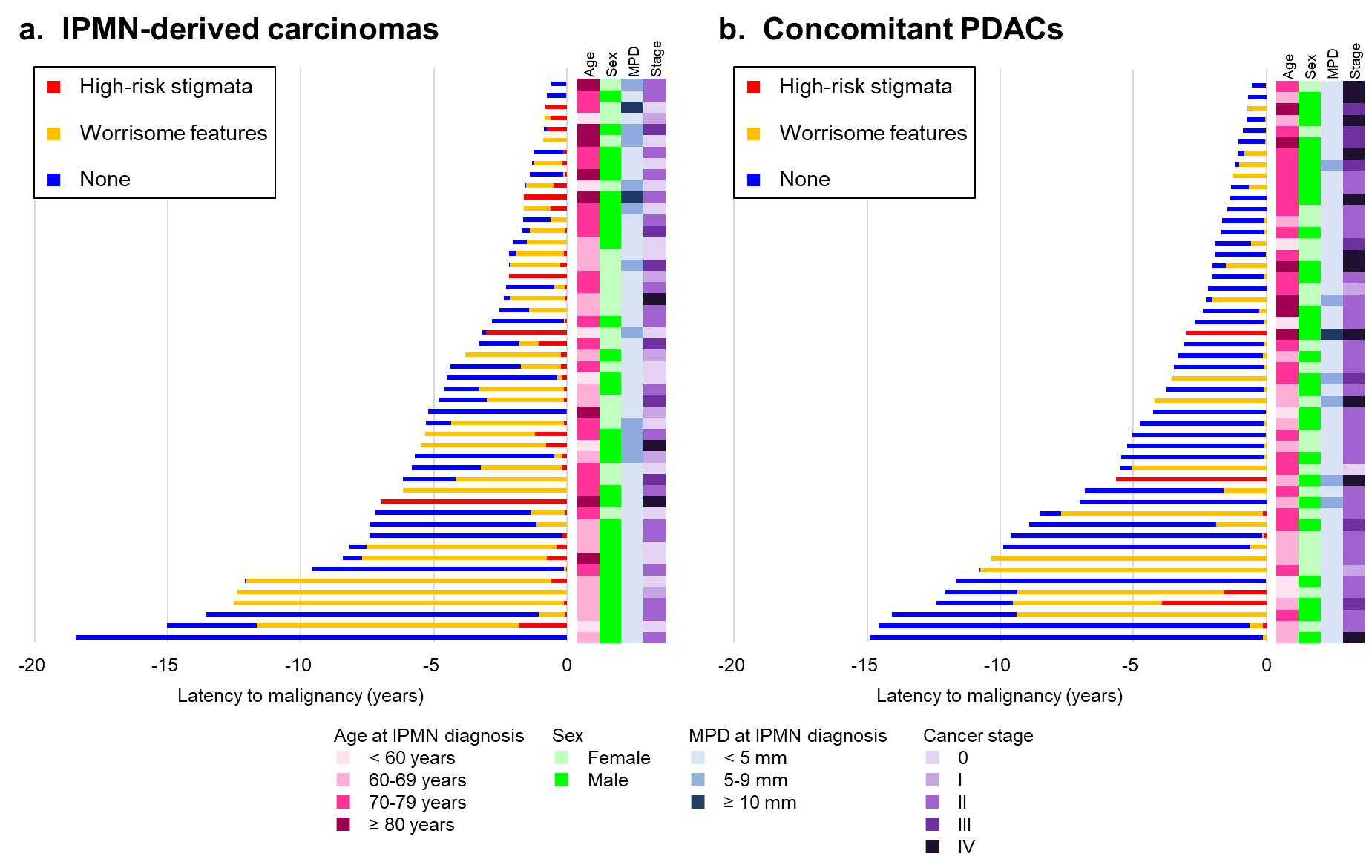
**

**Supplemenatary Fig. 5** Trajectory of morphologic features of IPMNs before pancreatic carcinoma diagnosis among patients with IPMNs, by carcinoma types. (a) IPMN-derived carcinomas (*n* = 50) and (b) concomitant PDACs (*n* = 50).

Abbreviations: IPMN, intraductal papillary mucinous neoplasm; MPD, main pancreatic duct; PDAC, pancreatic ductal adenocarcinoma.

**
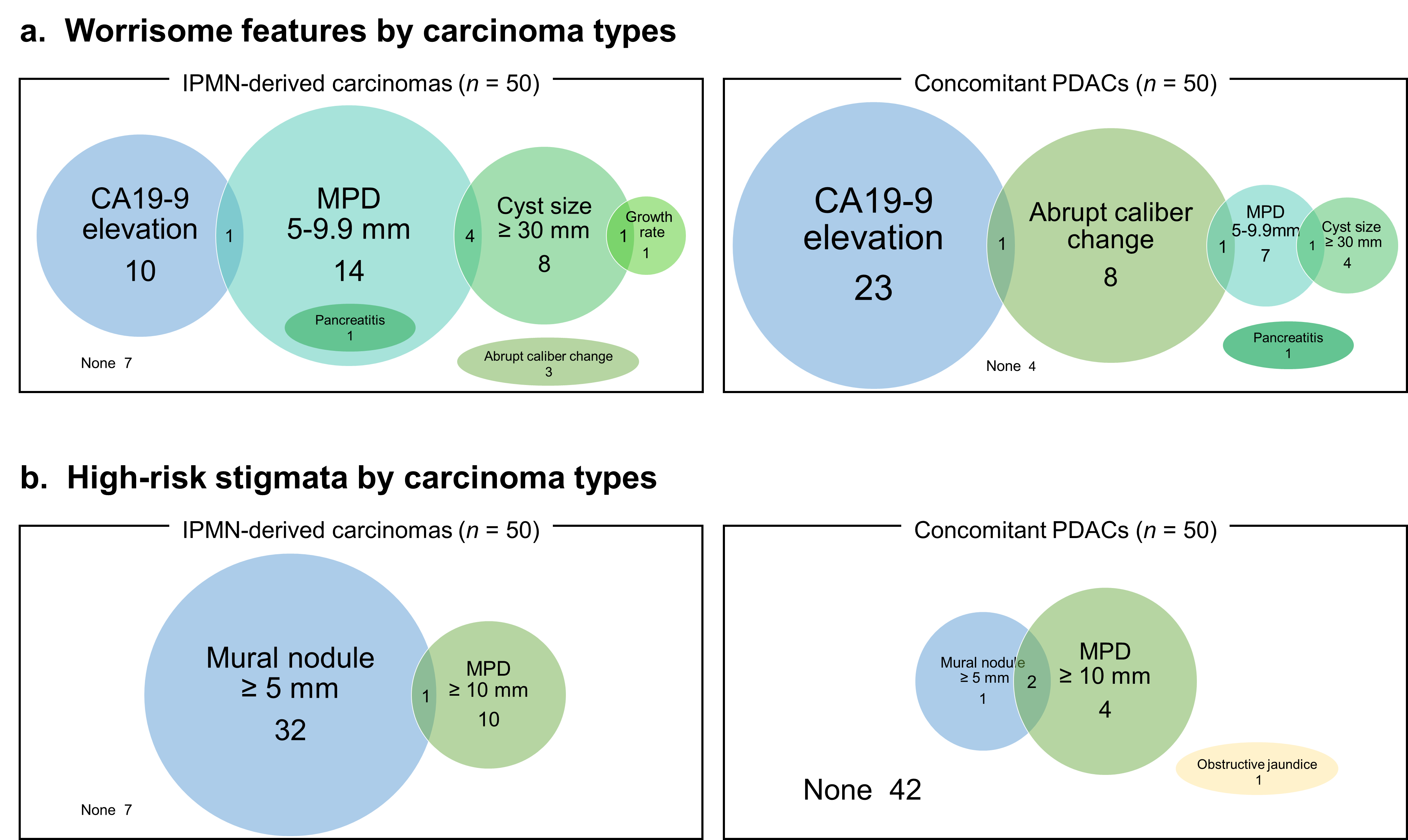
**

**Supplementary Fig. 6** Venn diagrams summarizing the types of worrisome features (a) and high-risk stigmata (b) observed during long-term surveillance of IPMNs, by carcinoma types. The first worrisome feature or high-risk stigma in a given case was considered.

Abbreviations: CA19-9, carbohydrate antigen 19-9; IPMN, intraductal papillary mucinous neoplasm; MPD, main pancreatic duct; PDAC, pancreatic ductal adenocarcinoma.

**Supplementary Table 1** Characteristics of patients diagnosed with pancreatic carcinomas during long-term surveillance of intraductal papillary mucinous neoplasms (IPMNs) at IPMN diagnosis

| Characteristic (*n* = 100)^a^ | |
| --- | --- |
| Age, years | 70.9 ± 8.5 |
|  |  |
| Sex |  |
| Male | 58 |
| Female | 42 |
|  |  |
| Year of IPMN diagnosis |  |
| 1994-2000 | 11 |
| 2001-2010 | 39 |
| 2011-2020 | 47 |
| 2021-2022 | 3 |
|  |  |
| HbA1c, % | 6.0 (4.5-10.0) |
|  |  |
| CA19-9, U/mL | 18 (1-730) |
|  |  |
| CEACAM5 (CEA), ng/mL | 3.7 (0.9-22.6) |
|  |  |
| Type of IPMN |  |
| Branch-duct type | 80 |
| Main-duct type | 6 |
| Mixed type | 14 |
|  |  |
| Location of IPMNs |  |
| Head | 28 |
| Body | 17 |
| Tail | 7 |
| Multifocal^b^ | 48 |
|  |  |
| Number of IPMNs |  |
| 1^b^ | 48 |
| 2-5 | 33 |
| ≥ 6 | 19 |
|  |  |
| Size of IPMN |  |
| < 10 mm | 22 |
| 10-19 mm | 34 |
| 20-29 mm | 26 |
| ≥ 30 mm | 12 |
| Not applicable^b^ | 6 |
|  |  |
| Diameter of the MPD |  |
| < 5 mm | 80 |
| 5-9.9 mm | 17 |
| ≥ 10 mm | 3 |
|  |  |

^a^ Data on continuous variables are presented as mean ± standard deviation or median (range).

^b^ These categories include main-duct IPMN cases with no cystic dilatation of branch ducts.

Abbreviations: CA19-9, carbohydrate antigen 19-9; CEA, carcinoembryonic antigen; HbA1c, hemoglobin A1c; IPMN, intraductal papillary mucinous neoplasm; MPD, main pancreatic duct.

**Supplementary Table 2** Diagnostic and treatment modalities for patients diagnosed with pancreatic carcinomas during long-term surveillance of intraductal papillary mucinous neoplasms

| Diagnostic modality | Pancreatic carcinoma cases (*n* = 100) |
| --- | --- |
| Pathological confirmation of malignancy |  |
| Surgery | 66 |
| EUS-FNA | 20 |
| ERCP | 5 |
| Biopsy of duodenal invasion | 1 |
|  |  |
| Clinical course | 8 |
|  |  |
| Treatment |  |
| Surgical resection | 65 |
| Pancreatoduodenectomy | 32 (49%) |
| Distal pancreatectomy | 33 (51%) |
|  |  |
| Neoadjuvant chemotherapy | 2 |
| Gemcitabine and nab-paclitaxel | 2 (100%) |
|  |  |
| Palliative chemotherapy | 20 |
| Gemcitabine | 10 (50%) |
| Gemcitabine and nab-paclitaxel | 3 (15%) |
| Gemcitabine and S-1 | 1 (5.0%) |
| S-1 | 1 (5.0%) |
| S-1, irinotecan, and oxaliplatin | 1 (5.0%) |
| Others | 3 (15%) |
| Unknown | 1 (5.0%) |
|  |  |
| Radiation | 1 |
|  |  |
| Best supportive care | 12 |
|  |  |

Abbreviations: ERCP, endoscopic retrograde cholangiopancreatography; EUS-FNA, endoscopic ultrasound fine-needle aspiration.

**Supplementary Table 3** Types of worrisome features and high-risk stigmata observed during long-term surveillance of intraductal papillary mucinous neoplasms and the timing of their occurrence, by carcinoma types

|  | Carcinoma type | |  |
| --- | --- | --- | --- |
|  | IPMN-derived  (*n* = 50) | Concomitant  (*n* = 50) | *P* value |
| **Worrisome features^a^** |  |  |  |
| Elevation of CA19-9 | 11 | 24 | 0.006 |
| Time to carcinoma,^b^ years | 1.2 (0.6-4.9) | 0.13 (0.04-0.67) |  |
|  |  |  |  |
| MPD = 5-9.9 mm | 20 | 9 | 0.015 |
| Time to carcinoma,^b^ years | 1.7 (1.1-3.4) | 1.2 (0.6-3.6) |  |
|  |  |  |  |
| Cyst size ≥ 30 mm | 13 | 5 | 0.037 |
| Time to carcinoma,^b^ years | 1.5 (1.1-4.2) | 9.4 (1.6-9.4) |  |
|  |  |  |  |
| Abrupt caliber change of MPD | 3 | 10 | 0.037 |
| Time to carcinoma,^b^ years | 0.12 (NA) | 0.08 (0.07-0.13) |  |
|  |  |  |  |
| Pancreatitis | 1 | 1 | 0.99 |
| Time to carcinoma,^b^ years | 1.5 (NA) | 0.01 (NA) |  |
|  |  |  |  |
| Cyst growth rate ≥ 5 mm/2 years | 2 | 0 | 0.15 |
| Time to carcinoma,^b^ years | 3.6 (NA) |  |  |
|  |  |  |  |
| Total | 43 | 46 | 0.34 |
| Time to carcinoma,^b^ years | 1.7 (0.9-4.0) | 0.2 (0.1-1.6) |  |
|  |  |  |  |
| **High-risk stigmata^a^** |  |  |  |
| Mural nodule ≥ 5 mm | 33 | 3 | < 0.001 |
| Time to carcinoma,^b^ years | 0.12 (0.08-0.23) | 0.1 (NA) |  |
|  |  |  |  |
| MPD ≥ 10 mm | 11 | 6 | 0.18 |
| Time to carcinoma,^b^ years | 0.8 (0.4-1.0) | 2.3 (0.5-3.7) |  |
|  |  |  |  |
| Obstructive jaundice | 0 | 1 | 0.31 |
| Time to carcinoma,^b^ years |  | 0.0 (NA) |  |
|  |  |  |  |
| Total | 43 | 8 | < 0.001 |
| Time to carcinoma,^b^ years | 0.2 (0.1-0.7) | 0.9 (0.1-3.2) |  |
|  |  |  |  |

^a^ A single case might be positive for multiple items that were observed at the same timing. The first worrisome feature or high-risk stigma in a given case was considered.

^b^ Data are shown as medians (interquartile ranges).

Abbreviations: CA19-9, carbohydrate antigen 19-9; IPMN, intraductal papillary mucinous neoplasm; MPD, main pancreatic duct; NA, not available.

**Supplementary Table 4** Types of worrisome features and high-risk stigmata observed at pancreatic carcinoma diagnosis, by carcinoma types

|  | Carcinoma type | |  |
| --- | --- | --- | --- |
|  | IPMN-derived  (*n* = 50) | Concomitant  (*n* = 50) | *P* value |
| **Worrisome features^a^** |  |  |  |
| Elevation of CA19-9 | 27 (54%) | 36 (72%) | 0.06 |
|  |  |  |  |
| Abrupt caliber change of MPD | 9 (18%) | 31 (62%) | < 0.001 |
|  |  |  |  |
| MPD = 5-9.9 mm | 21 (42%) | 14 (28%) | 0.14 |
|  |  |  |  |
| Cyst size ≥ 30 mm | 23 (46%) | 10 (20%) | 0.006 |
|  |  |  |  |
| Thickened cyst walls | 12 (24%) | 4 (8%) | 0.03 |
|  |  |  |  |
| Lymphadenopathy | 6 (12%) | 9 (18%) | 0.40 |
|  |  |  |  |
| Cyst growth rate ≥ 5 mm/2 years | 10 (20%) | 2 (4%) | 0.01 |
|  |  |  |  |
| Pancreatitis | 2 (4%) | 3 (6%) | 0.64 |
|  |  |  |  |
| Mural nodule < 5 mm | 1 (2%) | 0 | 0.31 |
|  |  |  |  |
| Any | 43 (86%) | 46 (92%) | 0.34 |
|  |  |  |  |
| **High-risk stigmata^a^** |  |  |  |
| Mural nodule ≥ 5 mm | 37 (74%) | 4 (8%) | < 0.001 |
|  |  |  |  |
| MPD ≥ 10 mm | 13 (26%) | 7 (14%) | 0.13 |
|  |  |  |  |
| Obstructive jaundice | 2 (4%) | 1 (2%) | 0.56 |
|  |  |  |  |
| Any | 43 (86%) | 8 (16%) | < 0.001 |
|  |  |  |  |

^a^ A single case might be positive for multiple items that were observed at the time of carcinoma diagnosis.

Abbreviations: CA19-9, carbohydrate antigen 19-9; IPMN, intraductal papillary mucinous neoplasm; MPD, main pancreatic duct.
